# Large-scale analysis of MYB genes in Cucurbitaceae identifies a novel gene regulating plant height

**DOI:** 10.1093/hr/uhaf210

**Published:** 2025-08-15

**Authors:** Wenxue Zhao, Jie Wang, Hongxue Yang, Xuyuan Hou, Zhonghua Zhang, Jiacai Chen, Huasen Wang, Chao Yan

**Affiliations:** Engineering Laboratory of Genetic Improvement of Horticultural Crops of Shandong Province, College of Horticulture, Qingdao Agricultural University, Qingdao 266109, China; College of Horticulture, Northwest A&F University, Yangling, Shaanxi 712100, China; Engineering Laboratory of Genetic Improvement of Horticultural Crops of Shandong Province, College of Horticulture, Qingdao Agricultural University, Qingdao 266109, China; College of Horticulture, Northwest A&F University, Yangling, Shaanxi 712100, China; Vegetable Crops, College of Horticulture, China Agricultural University, Beijing 100193, China; Engineering Laboratory of Genetic Improvement of Horticultural Crops of Shandong Province, College of Horticulture, Qingdao Agricultural University, Qingdao 266109, China; Engineering Laboratory of Genetic Improvement of Horticultural Crops of Shandong Province, College of Horticulture, Qingdao Agricultural University, Qingdao 266109, China; Engineering Laboratory of Genetic Improvement of Horticultural Crops of Shandong Province, College of Horticulture, Qingdao Agricultural University, Qingdao 266109, China; Engineering Laboratory of Genetic Improvement of Horticultural Crops of Shandong Province, College of Horticulture, Qingdao Agricultural University, Qingdao 266109, China; Engineering Laboratory of Genetic Improvement of Horticultural Crops of Shandong Province, College of Horticulture, Qingdao Agricultural University, Qingdao 266109, China

## Abstract

The MYB transcription factor (TF) family, which is involved in plant growth and development, is large and diverse. Previous studies on MYB family in Cucurbitaceae were mostly based on a single genome or focused on the R2R3 subfamily. Here, we analyzed 91 genomes of Cucurbitaceae and identified a total of 15 858 MYB genes. According to phylogenetic relationships, these genes were divided into 27 subgroups. The identified MYB genes were further classified into 121 MYB orthologous gene groups (OGGs), including 25 core, 57 softcore, 19 shell and 20 line-specific/cloud groups. Whole-genome duplication was the most common mechanism of MYB genes expansion. In core group, the higher proportions of MYB genes were found to be in the coexpression network constructed by the RNA-seq data. Through the comprehensive analysis including phylogeny and gene expression profile of cucumber MYB genes, as well as genetic variations in 103 cucumber germplasms, we identified a MYB gene *CsRAX5*, which may be related to cucumber plant height. We used gene editing technology to knockout and overexpress *CsRAX5*. In the knockout lines, *Csrax5*, the height was significantly increased compared with wild type (WT), whereas after overexpression the height of *CsRAX5-OE* plants was significantly decreased compared with WT. These results indicated that MYB gene *CsRAX5* negatively regulated cucumber plant height. The large-scale analysis of MYB genes in Cucurbitaceae in this study provides insights for further investigating the evolution and function of MYB genes in Cucurbitaceae crops.

## Introduction

Cucurbitaceae consists of 965 species in 95 genera [1]. It is the second largest fruit and vegetable subject after solanaceae [2]. Cucurbitaceous crops include cucumber (*Cucumis sativus*), watermelon (*Citrullus lanatus*), melon (*Cucumis melo*), pumpkin (*Cucurbita maxima*), and wax gourd (*Benincasa hispida*), etc. It is one of the most genetically diverse plant families whose members contain many health-promoting substances and play vital roles in sustaining human life [3, 4].

Transcription factors are characterized by the presence of distinct domains, including nuclear localization signaling domains, DNA-binding domains, transcriptional regulatory domains, and oligomerization domains [5]. Most transcription factors (TFs) can be classified into multiple subfamilies based on their encoded types of DNA-binding domains [6]. The MYB TF family is widely present in eukaryotes [7]. It is involved in the regulation of multiple biological processes, including plant growth and development, secondary metabolism, stress responses, and hormone signaling [8, 9]. MYB TFs are characterized by the presence of conserved DNA-binding structural domains at the N-terminus, including one or more repetitive R structures [10, 11]. Based on its structural characteristics, the MYB gene family can be divided into four types: R1, R2R3-MYB, R3-MYB, and R4-MYB, among which R2R3-MYB is the most common type and is widely present in plants [12]. MYB proteins typically contain one or more highly conserved MYB domains, each consisting of ~52 amino acids, forming three α helices, of which the second and third helices are involved in DNA binding [13]. In addition, MYB proteins also have a variety of C-terminal domains that are responsible for interacting with other proteins and regulating gene expression [14].

In recent years, the sequencing and assembly of Cucurbitaceae crop genomes have been gradually improved. Researchers selected 115 cucumber lines from 3342 germplasm resources for sequencing and genomic variation detection [15]. Subsequently, 11 germplasms from 115 cucumber lines were selected for chromosome-scale assembly, resulting in a map-based cucumber pangenome [16]. In watermelon, super-pan genomes were constructed using 547 germplasms to reveal variation in cultivated versus wild watermelon [17]. Subsequently, the telomere-to-telomere (T2T) genome of the *Citrullus* was assembled, covering 27 germplasms [18]. The assembly of melon T2T has also provided insights into understanding genome structure and molecular breeding [19, 20]. The pan-genome represents the variations of all genomes of a certain species. Compared with the traditional single reference genome, the pangenome can better reflect the variations within the species [21]. Based on a pangenome, structural variation can be compared and analyzed to reveal the evolutionary and domestication processes of Cucurbitaceae crops [22]. This provides assistance in mining key trait-related genes and studying variation in a particular gene family.

A genome-wide identification analysis revealed that genes in the same family usually had similar structures and motifs. Among the 130 *Arabidopsis* and 58 rice MYB genes identified, genes within the same subgroups exhibited conserved exon–intron structures [23]. A total of 175 MYB genes were identified in pumpkin and classified into 33 distinct subgroups (C1–C33), each with a highly conserved motif composition [24]. In total, 162 MYB genes have been identified from watermelon (*ClaMYB*); an amino acid alignment of all their MYB motifs shows a high degree of conservation [25]. Most current studies on the identification of the MYB genes in Cucurbitaceae focused on a single species, or only analyzed the R2R3 subfamily. Therefore, there is a lack of systematic identification and analysis of the MYB family in Cucurbitaceae. It is difficult to fully characterize the evolutionary history and conservation of the MYB gene family in Cucurbitaceae.

A great deal of researches have been done on MYB genes in regulating plant growth and development [26]. MYB24 can regulate stamen development [27] and MYB70 plays an important role in regulating seed germination and root development in *Arabidopsis thaliana* [28]. MYB37-encoded *RAX1* was shown to be transiently expressed in the stem tip meristematic tissue of Arabidopsis, regulating lateral branch formation [29]. In rice, MYB110 can regulate plant height, thereby affecting lodging resistance and grain yield [30]. MYB genes can trigger the formation of adventitious roots; additionally, the transcriptional cascade of *CsJAZ8* and *CsMYB6* responds to waterlogging by inhibiting ethylene and gibberellin accumulation, thereby triggering the formation of cucumber adventitious roots under flooding stress [31]. The MYB family member *CsRAXs* in cucumber regulates leaf size through auxin-mediated cell division, and mutations in *CsRAX1/2/5* promote leaf expansion, stem thickening, and fruiting ability enhancement [32]. The mapping and cloning of the plant height quantitative trait locus *qHT7.1* identified a MYB TF and revealed its role in controlling internode elongation, cell proliferation, and cell morphology in sorghum [33].

Plant height not only determines the morphological structure of the plant, but also closely related to the photosynthesis efficiency, ventilation, and light transmittance [34]. Cucurbitaceae plant height is an important agronomic trait affecting its growth, yield, and management [35, 36]. Therefore, it is of great significance to study plant height gene and its regulatory mechanisms to optimize cucumber cultivation management and improve yield [37, 38]. Most of the MYB genes identified in cucumber are involved in abiotic stress [39] and fruit pigmentation [40], etc. There are relatively few MYB genes regulating plant height, and further excavation of plant height-regulated genes is needed.

Here, we analyzed the phylogenetic relationships of MYB genes identified in the pangenome of Cucurbitaceae crops and investigated gene duplication events, coexpression networks, gene structure, and collinearity analysis. A plant height-related gene, *CsRAX5*, was also identified, and subsequent experiments showed that the gene negatively regulated cucumber plant height. These findings provide insights for understanding gene family analysis and studying the mechanisms of plant height regulation in cucumber .

## Results

### Identification and phylogenetic analysis of MYB genes in Cucurbitaceae crops

**Figure 1:**
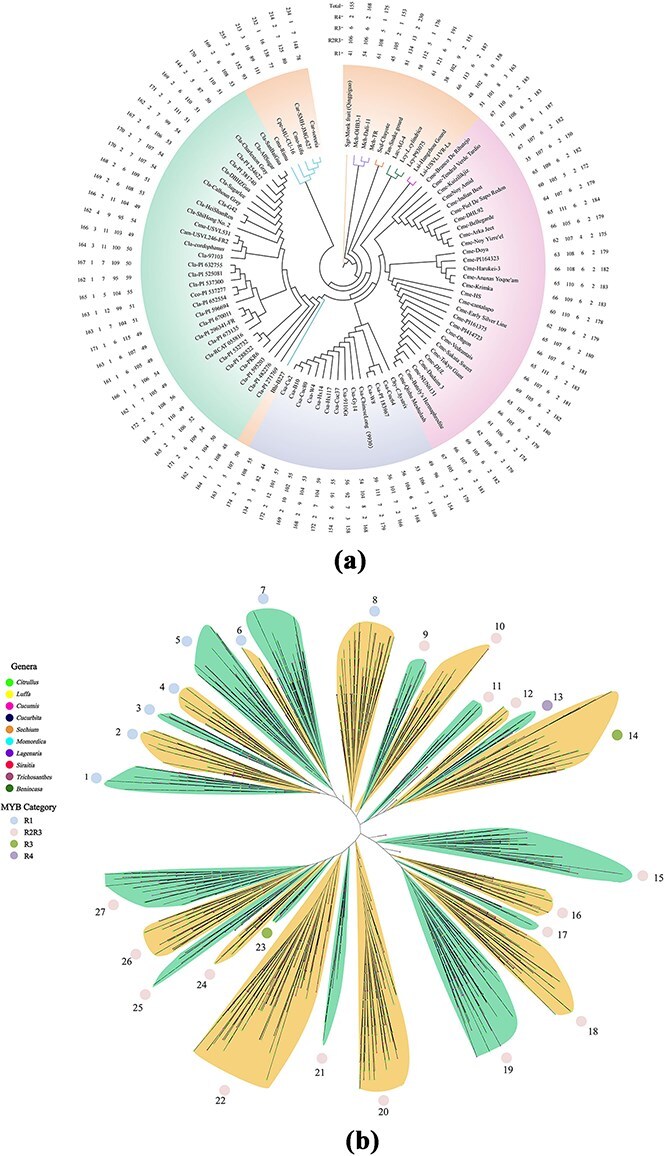
Identification of MYB genes in Cucurbitaceae accessions and phylogenetic analysis of MYB gene family. (a) The number of MYB genes identified in 91 accessions of Cucurbitaceae. The numbers from inner to outer represent the counts of R1, R2R3, R3, R4 and all MYBs, respectively. (b) Phylogenetic tree of 15 858 MYB genes identified in Cucurbitaceae which were divided into 27 branches, represented by number 1-27. Ten genera of Cucurbitaceae were assigned different color nodes at the end of phylogenetic branches.

A total of 15 858 MYB genes were identified in 91 genomes from 10 genera of Cucurbitaceae using combined BLASTP and HMMsearch approaches (Fig. 1a; Tables S1 and S2). A phylogenetic tree was constructed for these genes, which was divided into 27 subgroups according to their phylogenetic relationships. According to the number of MYB domains in each sequence, MYB genes were classified into four subfamilies: R1, R2R3, R3, and R4, which were mainly divided into 8, 16, 2, and 1 subgroups, respectively (Fig. 1b). A distribution pattern was observed among accessions: the R2R3 type (with dual MYB domains) dominated the MYB gene family, contrasting sharply with the rare occurrence of the R4 domain-containing members (Fig. 1a).

Our analysis revealed nonrandom distribution patterns of MYB genes across chromosomes, with significant heterogeneity observed both within individual chromosome and among different chromosomes within the same species. For example, in *Sechium edule*, the number of MYB genes is 36 on chromosome 5, whereas there are only four on chromosome 9 (Table S2). The observed gene distribution disparity could result from differential recombination activity across chromosomes, where chromosome 5’s position in a high-recombination zone promotes more frequent gene retention and duplication events.

### OGGs identification and gene duplication analysis for MYB genes of Cucurbitaceae crops

In order to overcome the limitation of a single reference genome, we utilized the collected 91 Cucurbitaceae genomes to determine the comprehensive evolutionary status of MYB gene family in Cucurbitaceae. Therefore, we took advantage of pangenome-based method to identify orthologous gene groups (OGGs) for MYB genes in all pangenome assemblies of Cucurbitaceae. The 15 858 MYB genes of the 91 genomes were grouped into 121 OGGs, including 25 core MYB groups (present in all 91 accessions), 57 softcore MYB groups (conserved in > 90% accessions), 19 shell MYB groups (10%-90% accessions) and 20 line-specific/cloud MYB groups (< 10% accessions) (Table S3).

**Figure 2:**
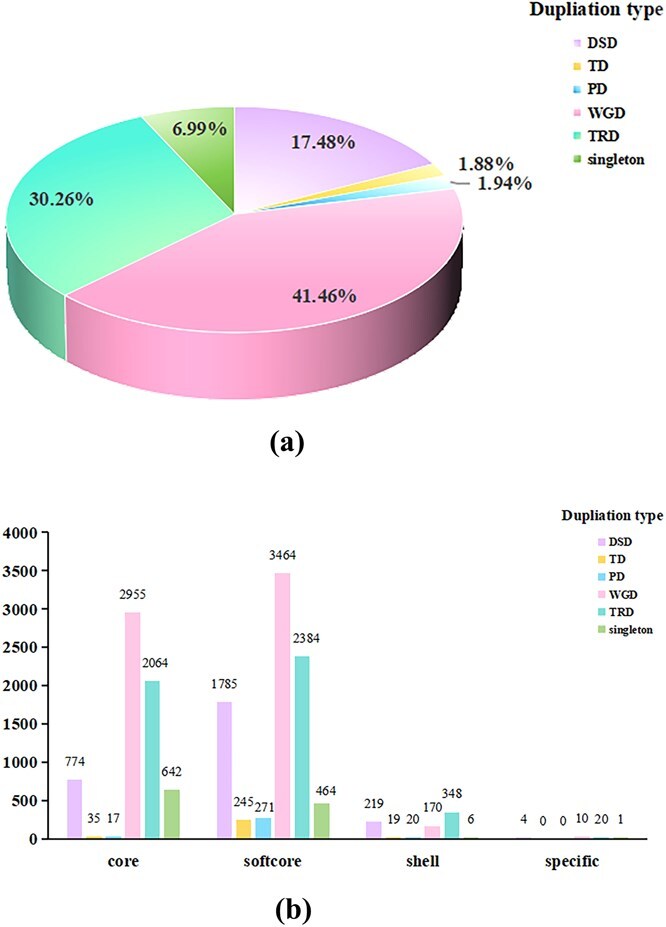
Gene duplication types identification and their distribution for core, softcore, shell, and specific MYB genes. (a) The proportion of different duplication types (WGD, TD, PD, TRD, DSD and singleton events) for Cucurbitaceae MYB genes. (b) The distribution of duplication modes for core, softcore, shell, specific MYB genes.

To investigate the mechanism for the expansion of MYB genes in Cucurbitaceae accessions, we analyzed gene duplication types including whole-genome duplication (WGD), transposed duplication (TRD), dispersed duplication (DSD), proximal duplication (PD) and tandem duplicate (TD) events. Among the 91 Cucurbitaceae accessions, WGD accounted for 41.46%, indicating it was the dominant mechanism for MYB genes expansion. In addition, TRD and DSD accounted for 30.26% and 17.48%, and PD and TD accounted for relatively small proportions, 1.94% and 1.88%, respectively (Fig. 2a).

Then, we counted the number of different duplication types (WGD, TRD, DSD, PD, TD) in core, softcore, shell, and line-specific/cloud genes. Analysis revealed distinct patterns of gene duplication among gene categories. Core genes showed 2955 WGD, 2064 TRD, 774 DSD, 17 PD, 35 TD events, 642 singletons. Softcore genes exhibited a larger number of WGD (3464), TRD (2394), DSD (1785), PD (271), TD (245) events, and singletons (464). The shell genes comprised 170 WGD, 348 TRD, 219 DSD, 20 PD, 19 TD events and 6 singletons. Notably, specific genes contained only 10 WGD, 20 TRD, 4 DSD events and 1 singleton, with complete absence of PD and TD events (Fig. 2b).

Compared with specific MYB genes and shell MYB genes, the distribution of WGD and TRD events in core MYB genes and softcore MYB genes is relatively large, indicating that core and softcore genes are mainly obtained due to WGD and TRD duplication. In shell MYB genes, the distribution of TRD duplication events is relatively large. However, in specific MYB genes, the number of various duplication events is relatively small. This indicates a correlation between the scale of gene duplication and the level of gene conservation [54].

### *Ka*, *Ks*, and *Ka/Ks* calculations and collinearity analyses

 The ratio of nonsynonymous substitution rates (*Ka*) to synonymous substitution rates (*Ks*), *Ka/Ks*, is a metric for detecting natural selection pressures. We utilized the *Ka/Ks* to determine the selection pressure on each OGG of Cucurbitaceae MYB genes. During the course of evolution, *Ka/Ks* > 1, *Ka/Ks* = 1, and *Ka/Ks* < 1 indicate positive selection, neutral selection and negative selection, respectively. 

**Figure 3:**
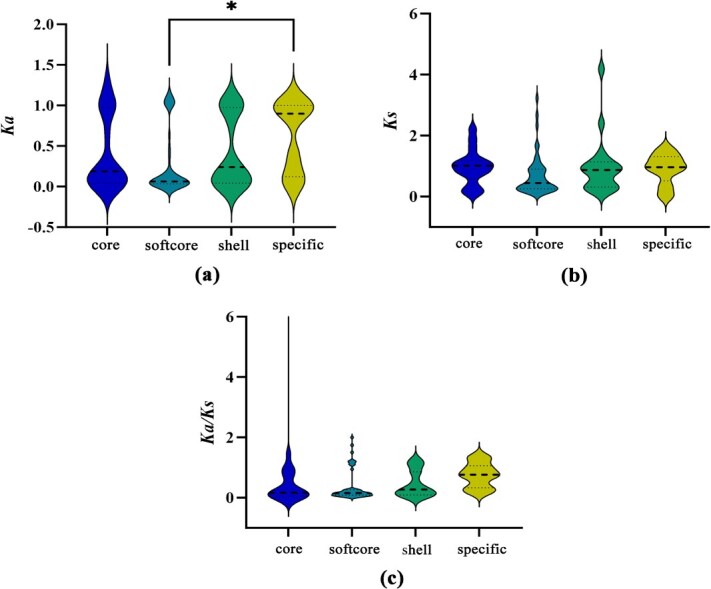
Analysis of natural selection on Cucurbitaceae MYB genes at the pangenome level. (a) *Ka* analysis of core, softcore, shell, and specific genes. (b) *Ks* analysis of core, softcore, shell, and specific genes. (c) *Ka/Ks* analysis of core, softcore, shell, and specific genes. Significance was analyzed using a one-way ANOVA test, *P* < 0.05.

Analyzing the *Ka* values revealed that the median value of the specific MYB genes is significantly higher than that of softcore (*P* < 0.05) genes, and the median values of the core and shell genes were similar (Fig. 3a); For the *Ks* and *Ka/Ks*, although there is no significant difference among these four categories, the median of specific MYB genes was higher relative to the other categories, indicating that specific genes generally experienced relaxed selection pressure (Fig. 3b; Fig. 3c). For *Ka/Ks*, the median values of the four categories were all relatively low, but extreme values occurred in the core genes. It indicates that the core genes were more likely positively selected to a greater extent compared with the other three categories (Fig. 3c).

The collinearity analysis serves as a powerful tool for elucidating mechanisms of genomic evolution and deciphering patterns of sequence conservation versus divergence across species. In this study, by analyzing MYB genes synteny among 11 Cucurbitaceae species, we identified extensive collinearity regions between closely related species, indicating their evolutionary conservation. For instance, cucumber and melon, chayote (*S. edule*) and snake gourd (*T. anguina*) both shared close phylogenetic relationships and correspondingly high collinearity regions (Fig. S1).

### Construction of MYB gene coexpression network

**Figure 4:**
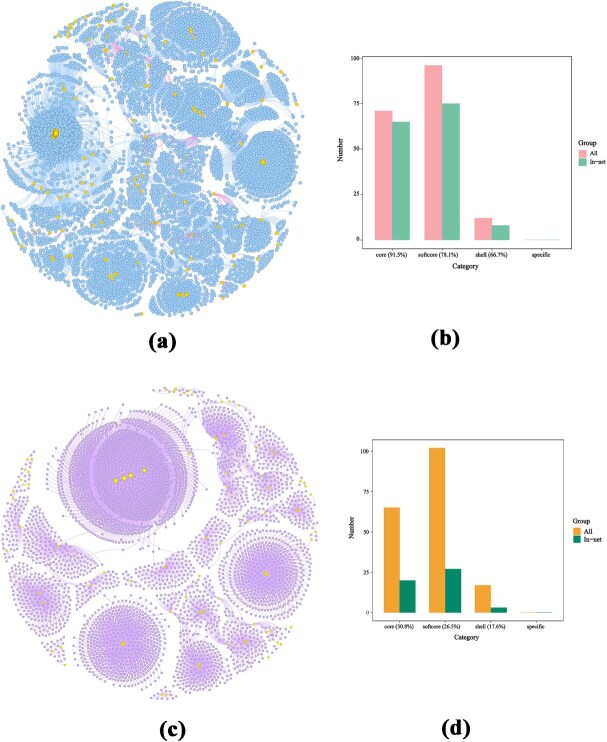
Coexpression networks of MYB genes constructed by the published RNA-seq data in cucumber and melon. (a) The coexpression network of cucumber MYB genes. (b) Core, softcore, shell and specific MYB genes in the cucumber coexpression network. (c) The coexpression network of melon MYB genes. (d) Core, softcore, shell and specific MYB genes in the melon coexpression network. The figure legend in both (b) and (d), ‘All’ represents all MYB genes in each category, ‘In-net’ represents MYB genes identified in the network. The ratios in parentheses (core, softcore, shell) indicate the proportion of ‘In-net’ genes relative to ‘All’ genes in each category.

Using the published RNA-seq data of 10 different tissues from cucumber (*C. sativus*) [41] and 45 different tissues or developmental stages from melon (*C. melo*) [42], the PCC (Pearson correlation coefficient) were set as >0.95 or <−0.95, and the coexpression networks of 179 MYB genes in cucumber and 184 MYB genes in melon were constructed. In the cucumber coexpression network, a total of 6760 genes showed significant correlation with the 148 MYB genes, comprising 6532 positively correlated and 241 negatively correlated genes (Fig. 4a). *CsaV3_1G031310* had the largest number of correlative genes (1280) (Table S4). In the melon coexpression network, there were 2540 genes correlated with 50 MYB genes. All these 2540 genes were positively related to MYB genes (Fig. 4c; Table S5). And we counted the number of genes correlated with each MYB gene in the four categories (core, softcore, shell and specific) in the cucumber and melon coexpression network, and there was no significant difference among the four categories (Fig. S2).

In the coexpression network of cucumber MYB genes (Fig. 4a), 65 core, 75 softcore, 8 shell and none specific MYB genes were discovered (Fig. 4b). It accounted for 91.5% of all core MYB genes, 78.1% of all softcore MYB genes, and 66.7% of all shell MYB genes. Among them, core genes accounted for the highest proportion, while no specific genes were identified (Fig. 4b). In the coexpression network of melon MYB genes, 20 core, 27 softcore, 3 shell and none specific MYB genes were discovered (Fig. 4c; Fig. 4d). It accounted for 30.8% of the total core genes, 26.5% of all softcore genes, and 17.6% of all shell genes (Fig. 4d). Overall, we found that core MYB genes in the networks accounted for the highest proportion in both cucumber and melon, followed by softcore genes and shell genes, with no specific genes identified.

### Gene structure and motif analyses of MYB gene family of Cucurbitaceae

We performed a structural analysis of the MYB genes in Cucurbitaceae and counted the number of exons (Table S6). The number of exons with 3 in each species was the greatest, and most of the gene structures is manifested as three exons and two introns, which indicates that this is a relatively common gene structure pattern. Some of the Cucurbitaceae species showed multiple exons, and in chayote (*S. edule*) and snake gourd (*T. anguina*), genes containing more than 3 exons account for a higher proportion compared to other Cucurbitaceae, indicating the increased complexity of gene function in these two species. Multiple exons usually indicate that more mRNA and protein isomers have been produced by alternative splicing. In addition, closely related species, such as cucumber and melon, had similar genetic structures and roughly the same number of exons, suggesting that they are evolutionarily conserved. In addition, about 70% of *CsaMYB* and *CmaMYB* genes had no more than three introns. Differences in genetic structures between species can reflect species-specific characteristics.

**Figure 5:**
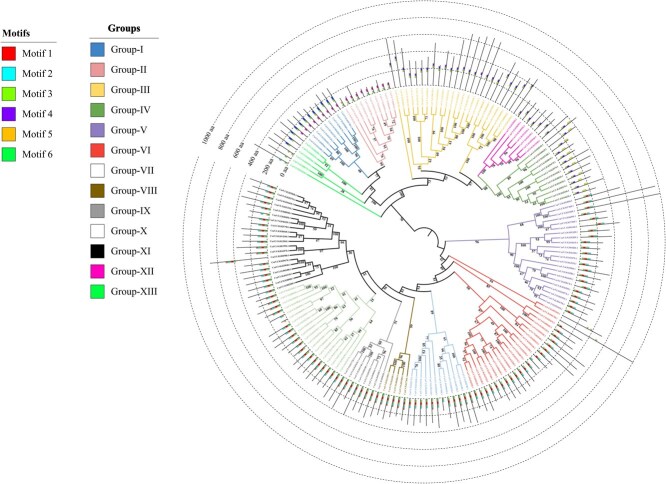
The evolutionary relationships and motif distributions of the MYB gene in cucumber. Motifs, numbered 1-6 are represented by boxes of different colors. The phylogenetic tree is divided into 13 groups, each distinguished by a different color.

Motif similarity may be due to the same or similar TFs recognizing and binding to sequences in different species. In the same subfamily, different cucurbit species had similar motif structures, which indicates that these motifs play important conserved roles in regulating gene expression (Fig. S3). In closely related Cucurbitaceae species, the motif structures were generally similar. For example, the motif structures and numbers in cucumber and melon were roughly similar. The motifs of *B. hispida* and *L. siceraria* were roughly similar, and those of *T. anguina* and *S. edule* were similar. Cucumber MYB genes were divided into 13 branches based on the evolutionary relationships, and the motifs in each branch were roughly consistent (Fig. 5). In some specific cases, motifs may also exhibit certain variations despite high conservation within the same branch, and this specificity may be related to the species’ environmental adaptation.

### A *CsMYB* gene, *CsRAX5*, regulates cucumber plant height

Stem branch length is a major determinant of plant height. Elongation of the aboveground portion is mainly influenced by the apical meristematic organization of the stem tip [43]. Keller *et al*. [29] found that *RAX1* encoded by MYB37 is transiently expressed in the stem tip meristem of *A. thaliana*. In the phylogenetic tree of *A. thaliana* and cucumber, MYB37 was located in the S2 branch, and a homologous gene of MYB37 was identified. We found that *Csav3_UNG046320* (*CsRAX5*) belonged to the same subfamily with MYB37 (Fig. S4).

It was found in previous studies that *CsRAX5* negatively regulates leaf size [32]. By analyzing the expression levels of 10 genes in each tissue of the S2 branch, the expression of *CsRAX5* was found to be relatively low in the apical meristematic tissue. Through a comprehensive analysis, it was discovered that *CsRAX5* was lowly expressed in the tissue of young and adult stems (Table S7). Thus, we hypothesized that *CsRAX5* has a negative regulatory effect on plant height.

**Figure 6:**
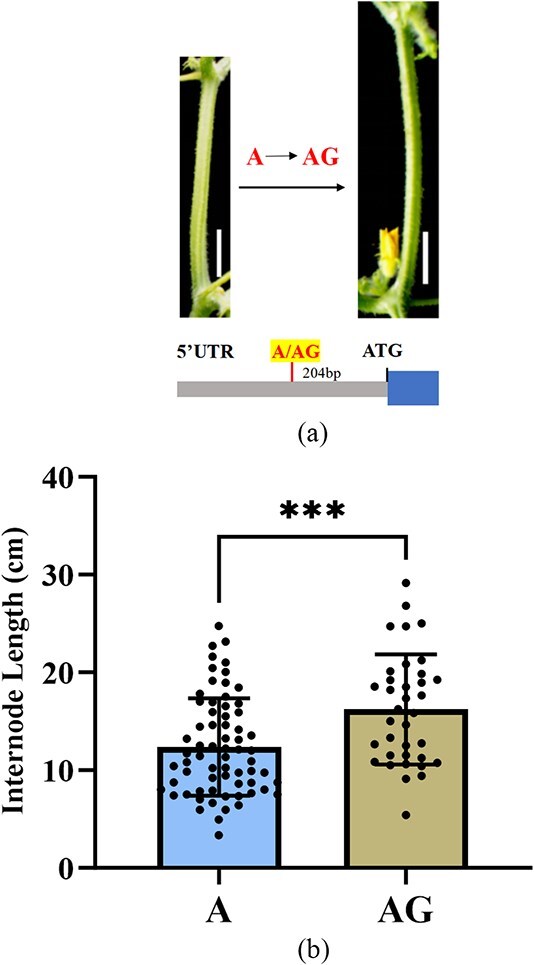
Correlation of differences in internode length at the same node position in cucumber with *Csrax5* variation among 103 cucumber accessions. (a) The internode length phenotype in cucumber accessions. Left, short internode phenotype; Right, long internode phenotype. Bar = 1 cm. The models at the bottom indicate the mutation sites in the promoter region of the gene *CsRAX5*. (b) Internode length of plants with different genotypes in 103 cucumber accessions.

An analysis of 103 cucumber germplasms from a natural population showed that there were base mutations in the promoter region of gene *CsRAX5*. In the natural population, plants with an A base at 204 bp prior to the ATG start site showed short internodes, whereas plants with AG bases at the same position showed long internodes (Fig. 6a and b).

### Knockout of *CsRAX5* in cucumber increases plant height

**Figure 7:**
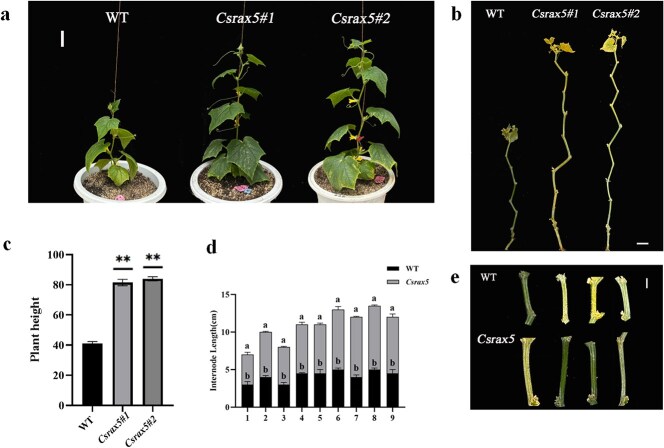
Phenotypes of cucumber plants in WT and *CsRAX5* knockout lines. (a) The phenotypes of cucumber plants in WT and knockout lines, where WT represents the wild type (‘R1461’), and ‘*Csrax5#1*’ and ‘*Csrax5#2*’ represent the two knockout lines. Bar = 5 cm. (b) The internode phenotype of the plants in WT and knockout lines. Bar = 5 cm. (c) The heights of WT and knockout lines in centimeters. (d) The internode lengths of WT and knockout lines. (e) The single internode length phenotypes of WT and knockout lines. Bar = 1 cm.

To experimentally validate the hypothesis that *CsRAX5* negatively regulates cucumber plant height, we obtained ‘*Csrax5#1*’ and ‘*Csrax5#2*’ lines after gene editing using CRISPR/Cas9 technology, and found that the *Csrax5* mutants were significantly higher than wild type (WT). After knockout, the average plant height was ~80 cm, which was twice that of the WT (Fig. 7a and c). Moreover, the internode length of *Csrax5* mutant was significantly greater than that of WT (Fig. 7b, d, and  e), and the internode length increased by ~1–2 cm. The above experimental data indicate that *CsRAX5* negatively regulates cucumber plant height.

### Overexpression of *CsRAX5* reduces plant height in cucumber

**Figure 8:**
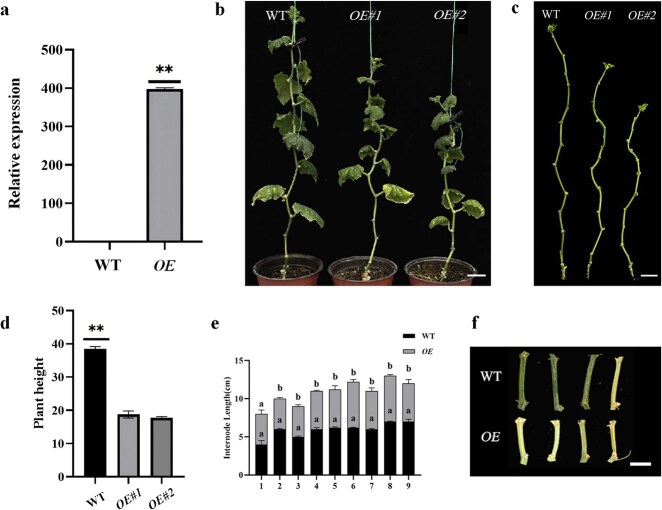
Phenotypes of cucumber plants in WT and *CsRAX5* overexpression lines. (a) The expression level of ‘*CsRAX5-OE*’ after *CsRAX5* overexpression, with ‘**’ indicating an extremely significant level (*P* < 0.01). (b) The phenotypes of cucumber plants in WT and overexpression lines, WT represents the wild type (‘XTMC’), and ‘*OE#1*’ and ‘*OE#2*’ represent two overexpression lines. Bar = 5 cm. (c) The internode length of plant in WT and overexpression lines. Bar = 5 cm. (d) The heights of WT and overexpressed lines. (e) Statistics of internode length in WT and overexpression lines. (f) Internode phenotypes of WT and overexpression lines. Bar = 1 cm.

To further explore the function of *CsRAX5*, we constructed overexpression plants of *CsRAX5* driven by the cauliflower mosaic virus (CaMV) 35S promoter and obtained overexpressed lines ‘*CsRAX5-OE#1* (*OE#1*)’ and ‘*CsRAX5-OE#2* (*OE#2*)’. Quantitative real-time polymerase chain reaction (RT-qPCR) of the overexpression lines revealed that *CsRAX5* expression was significant (*P* < 0.01) higher than that of the WT (Fig. 8a). After overexpressing, plant heights were significantly shorter than those of WT. The average plant height was ~20 cm, which was 40% shorter than that of the WT (Fig. 8b and d). Additionally, the internode lengths of the ‘*OE#1*’ and ‘*OE#2*’ overexpression lines were significantly shorter than those of the WT (Fig. 8c, e, and  f), with a reduction of ~2 cm compared to the control. Thus, after the overexpression of *CsRAX5*, cucumber plant grew slowly and the plant height decreased significantly.

## Discussion

The MYB gene family, widely existing in eukaryotes, plays important roles in plant growth and development, secondary metabolism, and stress responses [44]. In cucumber, *CsMYB36* regulates peel color [45]. Besides, *CsMYB36* plays an important role in cell proliferation, with shortened fruit necks occurring after its overexpression in cucumber *ygp* mutants [46]. Using a transcriptome, the previous study found that the expression of MYB genes related to cucumber peel development increased at 3, 6, and 9 days compared with the control [47]. Researchers identified 55 genes in the R2R3 subgroup of cucumber MYB genes, which were divided into 11 subfamilies based on phylogenetic relationships, and found that some of these genes are involved in abiotic stress (NaCl, low temperature, and abscisic acid) responses through a transcriptional analysis [48]. In this study, through knockout and overexpression experiments, we found that the MYB gene *CsRAX5* plays an important role in controlling plant height.

A single linear reference genome has certain limitations, which may lead to reference bias and affect the detection of genetic variation within species [49]. Moreover, pan-genomic work is more limited due to the lack of integrated comparative genomic tools [50]. For this reason, researchers have specifically integrated comprehensive databases for analysis [51–53]. Tong *et al*. [54] identified bHLH gene family using pan-genome data from 20 barley varieties and divided them into 201 OGGs. They also proposed an innovative pan-genomic-based analysis method. Researchers analyzed 61 tomato varieties based on a super pan-genome and revealed 59 066 orthologous groups [55]. At present, T2T reference genomes are gradually improving the quality of single linear genomes, and the pan-genome plays an important role in the study of genome complexity, crop origin, and domestication, as well as the and genetic-based improvement of agronomic traits. In this study, publishing pan-genomic data of Cucurbitaceae were integrated to identify MYB family genes, covering 91 accessions of 10 genera and providing a reference for the analysis of genetic diversity and evolutionary relationships among different species. Meanwhile, our pan-genomic research method can be extended to other gene families, such as WRKY and NAC etc, and through the OGGs analysis, conserved and specific genes can be identified, which is conducive to the discovery of highly specific genes and fast-evolving genes among pan-genome of Cucurbitaceae crops.

Plant height is a key agronomic trait that significantly influences crop yield, plant architecture, and environmental adaptability [35]. Moderate dwarfism is a valuable agronomic trait in modern agriculture because it can reduce plant toppling and increase the harvest index [37]. A large number of plant height-related MYB genes have been identified, and in rice, overexpression of the MYB gene *OsMPH1* resulted in higher plants [56]. In soybean, overexpression of the *GmGAMYB* gene increased plant height [57]. In this study, we observed that *CsRAX5* knockout plants exhibited increased plant height compared to WT. However, after its overexpression, the plants were dwarfed and the internode length was shortened compared with WT. These results indicate that this gene plays a key role in controlling plant height.

WGD generally plays an important role in species formation, adaptation, and diversification [58]. WGD is the dominant duplication mechanism and is essential for maintaining the core functions shared by the species. Approximately 74% of lipid synthesis-related genes originate from WGD events in *Spermophilus oleifera* [59]. In this study, we found that WGD represented the highest percentage, at 41.46%, of duplication events in the pan-genome. It is the central driver for gene evolution in cucurbit crops. PD and TD accounted for a low percentages and hardly contributed to the core genes. But both duplication events are important for genomic structural variation. TD can drive adaptive evolution, and TD events in the *NRAMP3* gene of *Populus* spp. result in copied gene sequence variations that give rise to functional divergence [60]. TD and PD have important roles in the ginsenoside biosynthetic genes and contribute to their diversification [61].

In this study, we identified *CsRAX5* as a novel plant height regulator, which is a divergence from the canonical *RAX* function in leaf axil initiation [29, 62, 63]. In the study of Chen *et al*. [32] *CsTPST*, *CsBRC2*, and *CsCMI1* may be the downstream genes of *CsRAX*. In the future, we will search for interacting genes using transcriptome data and DAP-seq [64], and we will validate the possible downstream genes using yeast single-hybrid and dual-luciferase assays.

## Materials and methods

### Data collection and MYB gene identification in Cucurbitaceae genomes

Genome sequence data of 91 accessions were obtained from the database as described in Table S1. We obtained the genomic sequence files (Genome), amino acid sequence files (PEP), and gene annotation files (GFF3 files). Among them, the cucumber data were obtained from http://cucurbitgenomics.org/v2/ and http://www.cucumberdb.com/; Watermelon data were obtained from http://cucurbitgenomics.org/v2/ and http://www.watermelondb.cn; Melon data were obtained from http://cucurbitgenomics.org/v2/, https://www.ncbi.nlm.nih.gov/, and https://ngdc.cncb.ac.cn; Genome sequence data of the other Cucurbitaceae species were obtained from http://cucurbitgenomics.org/v2/.  *Arabidopsis thaliana* MYB genome sequences, amino acid sequences, and gene annotation files were from https://www.arabidopsis.org. Markov model (HMM) profile PF00249 was used as queries to identify MYB proteins from Cucurbitaceae genomes by HMMER software (https://www.ebi.ac.uk/Tools/hmmer/). The MYB protein sequences of *A. thaliana* were used as queries for BLASTP search 2.15.0 (https://blast.ncbi.nlm.nih.gov/Blast.cgi). The genes jointly identified by the two methods were utilized as candidate MYB genes and manually corrected to remove redundant protein sequences using Interpro (https://www.ebi.ac.uk/interpro/).

### Phylogenetic tree construction

OrthoFinder software [65] was used to identify single-copy genes from 91 accessions, and a phylogenetic tree was constructed using these genes. Sequence alignment was performed using MAFFT software (http://mafft.cbrc.jp/alignment/software/), and a phylogenetic tree was constructed using IQ-TREE2 v2.2.2.6 (http://www.iqtree.org/) with the Maximum Likelihood method. The phylogenetic tree was validated using 1000 bootstrap replicates. The phylogenetic tree of 15 858 MYB genes was constructed using FastTree (v2.1.11 http://www.microbesonline.org/fasttree/) under the Maximum Likelihood method. Branch supports were evaluated by Shimodaira-Hasegawa-like (SH-like) test with 1,000 likelihood resamples. The phylogenetic trees of MYBs were classified into subfamilies and visualized using iTOL (https://itol.embl.de/personal_page.cgi).

### Duplication events and the identification of OGGs

DupGen_finder [66] was used to identify the gene duplication types including WGD, TD, PD, TRD, and DSD. Using the protein sequences of the grape genome (GCF_030704535.1) as an outgroup, a Blastp analysis was performed on the Cucurbitaceae pan-genome.

OrthoFinder [66] was used to analyze core, softcore, shell, and specific genes. The MYB protein sequences from all accessions and the resulting Orthogroups file was used to classify and evaluate these genes according to the following criteria: core (100% in all accessions), softcore (>90% of accessions), shell (10%–90% of accessions), and specific (<10% of accessions) genes.

### *Ka*, *Ks*, and *Ka/Ks* analysis

The ParaAT tool [67] was used to calculate *Ka*, *Ks*, and *Ka/Ks* value of homologous MYB gene pairs in the Cucurbitaceae pan-genome, with the alignment parameters set to the default values of the MAFFT software. Violin plots for *Ka*, *Ks*, and the *Ka/Ks* ratio were constructed using GraphPad Prism 10. Significance analysis was determined by a one-way analysis of variance, with a *P*-value <0.05 regarded as significant.

### Coexpression network construction for MYB genes

We used published RNA-seq data of 10 different tissues from cucumber (*C. sativus*) [41], and 45 different tissues or developmental stages from melon (*C. melo*) [42]. The Pearson correlation coefficient was employed to build a proximity matrix, and a coexpression network was constructed based on the corresponding correlation matrix. When |PCC| > 0.95 [61], the MYB genes served as the core nodes of the coexpression network, with target genes acting as secondary nodes, to establish the relevant networks for cucumber and melon. Genes with an absolute correlation value exceeding 95% were selected. The coexpression network was visualized using Gephi (v0.8.2).

### Gene structure and protein motif analysis

To obtain visual information on gene structures, the GFF3 files of MYB genes were uploaded to the online website GSDS. The MYB protein sequences from Cucurbitaceae species were analyzed using the online tool MEME Suite with default parameters (https://meme-suite.org/meme/) to explore the motifs of members of this gene family. The obtained MEME HTML file and treefile were imported into iTOL to draw the combined diagram of the phylogenetic tree and motifs.

### Collinearity analysis of the MYB gene in Cucurbitaceae crops

To visually illustrate the conserved regions and homologous relationships among genomes, we performed a collinearity analysis on 11 Cucurbitaceae species using TBtools software. Genomic files, GFF3 annotation files, and target gene IDs of each species were collected. Comprehensive analysis and visualization were conducted with TBtools, where the layout was carefully adjusted and a color-coding scheme with distinct hues was employed to clearly delineate syntenic relationships across different species.

### Genetic transformation of the *CsRAX5* in cucumber

We employed a dual-target knockout strategy to perform genetic transformation in cucumber, resulting in the generation of *CsRAX5* knockout lines. The sgRNA target was designed using the online website CRISPR direct (https://crispr.dbcls.jp/). The target site was then constructed into the pKSE402 vector to form the knockout vector [68]. The coding DNA sequence was constructed into intermediate vector Puc19-cflag, and the CsRAXs-cflag fragment was constructed into the overexpression vector pCAMBIA1305 [68]. Each constructed vector was sequenced to ensure the accuracy of the sequence, and Agrobacterium-mediated transformations were carried out using line GV3101. The *CsRAX5* gene knockout vector was transformed into ‘R1461’ as the background material, and the overexpression vector was transformed into ‘XTMC’. The genetic transformation vectors each carried the GFP tag.

### Plant materials and growth conditions

The cucumber materials including ‘XTMC’, ‘R1461’, ‘*Csrax5*’ (knockout lines), and ‘*CsRAX5-OE*’ (overexpression lines) were cultivated in the greenhouse of Northwest A&F University with sufficient water, fertilizer, and light. Plant height and internode length were measured at 5-day interval from the onset of flowering. Cucumber apical bud tissue from the growth period were collected and immediately frozen in liquid nitrogen and stored at −80°C. These materials were used for RNA extraction and RT-qPCR analysis.

### RNA extraction and real-time quantitative PCR

Total RNA were extracted from cucumber apical bud tissue of ‘*Csrax5*’, ‘*CsRAX5-OE*’, ‘XTMC’, and ‘R1461’ using a Vazyme RNA extraction kit (FastPure Universal Plant Total RNA Isolation Kit). A Vazyme reverse transcription kit was used to reverse transcribe 1 μg of each RNA sample. The reaction procedure was as follows: after 15 min at 50°C and 5 s at 85°C, the MonAmp™ SYBR Green qPCR Mix was used to configure an RT-qPCR system of 20 μl, and fluorescence quantitative detection was performed on a 96-well plate with three biological replicates per sample. The relative gene expression of *CsRAX5* was calculated by the 2^-∆∆Ct^ method. Prime premier 5.0 software was used to design the primers (Table S8), and the cucumber housekeeping gene *CsUBI* [32] was selected as the internal reference gene.

### Statistical analysis

The experiments contained three biological replicates, each consisting of five plants. After measuring the heights of the plants, the average value was calculated. The same section was selected for internode length measurements. All the data were analyzed using SPSS software. The significance was determined using Student’s *t*-test at *P* < 0.05.

## Supplementary Material

Web_Material_uhaf210

## Data Availability

All data supporting the results of this study are included in the article and additional files.
