# Supplementary material for: Large-scale analysis of MYB genes in Cucurbitaceae identifies a novel gene regulating plant height
Source: Hortic Res. 2025 Aug 15;12(11):uhaf210. doi: 10.1093/hr/uhaf210 (PMC12578468; doi:10.1093/hr/uhaf210)
Supplement: Web_Material_uhaf210 [file web_material_uhaf210.zip › Figure S1.pdf]

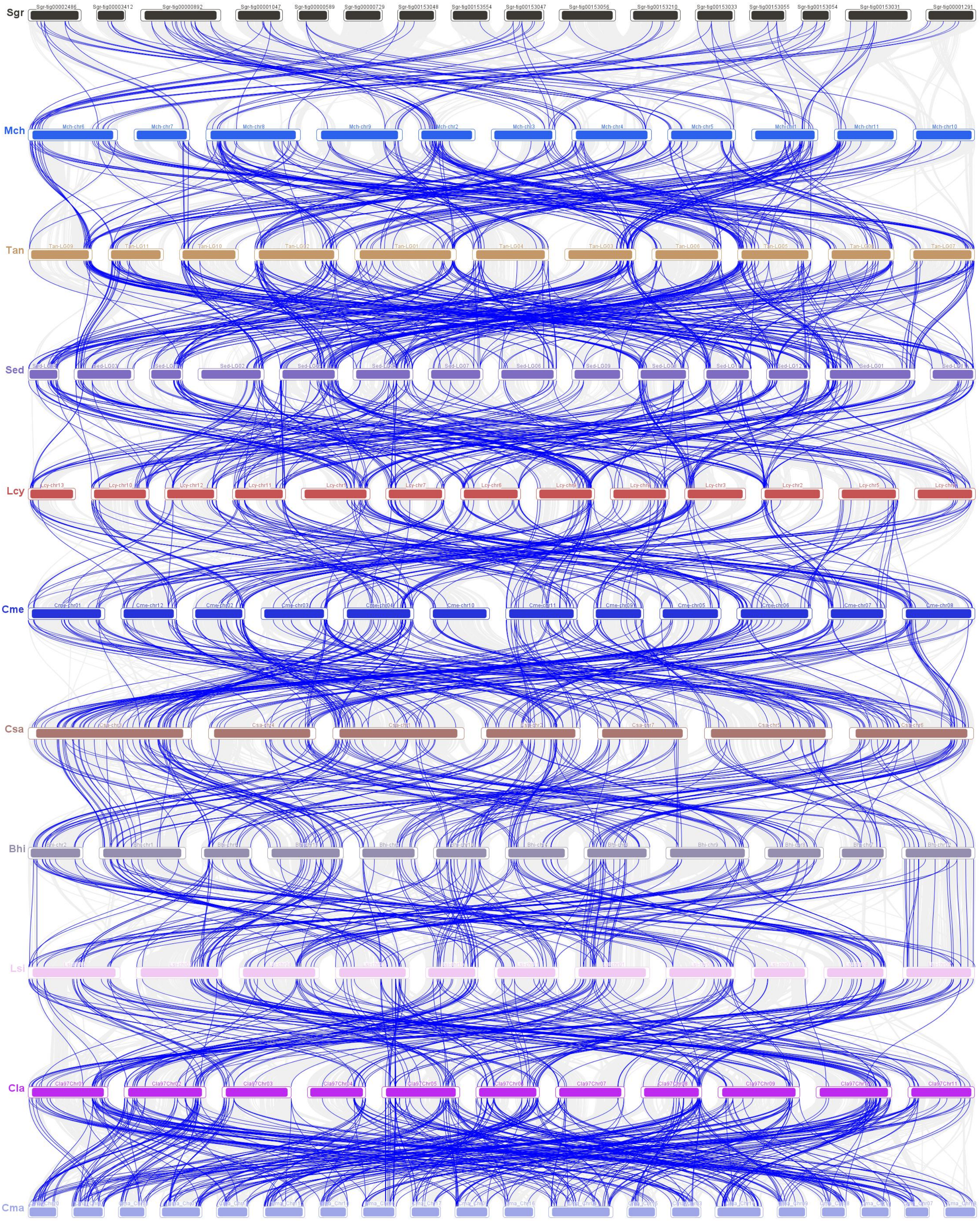

Supplementary figure 1. Collinearity analysis of MYB genes in 11 Cucurbitaceae species. Blue-highlighted lines represent MYB orthologous genes, while gray lines indicate homologous genes across the 11 Cucurbitaceae species (Mch-*Momordica charantia*, Lis -*Lagenaria siceraria*, Bhi-*Benincasa hispida*, Cla-*Citrullus lanatus*, Cme-*Cucumis melo*, Csa-*Cucumis sativus*, Cma-*Cucurbita maxima*, Sgr-*Siraitia grosvenorii*, Lcy-*Luffa cylindrica,* Sed-*Sechium edule*, Tan-*Trichosanthes anguina*).
