# Supplementary material for: Large-scale analysis of MYB genes in Cucurbitaceae identifies a novel gene regulating plant height
Source: Hortic Res. 2025 Aug 15;12(11):uhaf210. doi: 10.1093/hr/uhaf210 (PMC12578468; doi:10.1093/hr/uhaf210)
Supplement: Web_Material_uhaf210 [file web_material_uhaf210.zip › Figure S2.pdf]

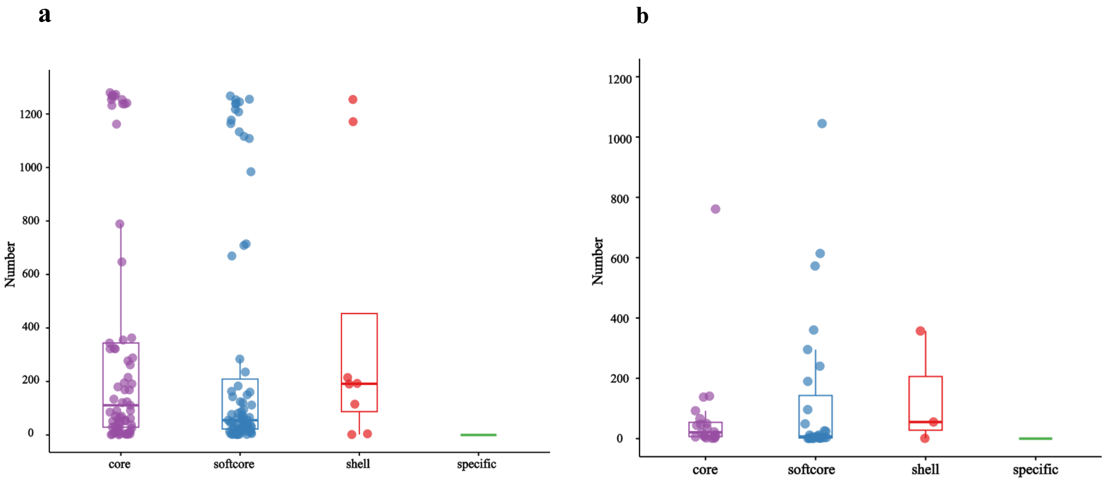

Supplementary figure 2. The number of genes co-expressed with each MYB gene in *Cucumis sativus* (a) and *Cucumis melo* (b).
