# Supplementary material for: Large-scale analysis of MYB genes in Cucurbitaceae identifies a novel gene regulating plant height
Source: Hortic Res. 2025 Aug 15;12(11):uhaf210. doi: 10.1093/hr/uhaf210 (PMC12578468; doi:10.1093/hr/uhaf210)
Supplement: Web_Material_uhaf210 [file web_material_uhaf210.zip › Figure S3.pdf]

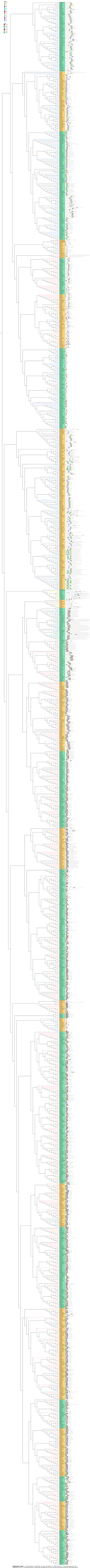

Supplementary Figure 3 . The phylogenetic relationships and motif distributions of MYB proteins in 11 Cucurbitaceae species. The phylogenetic tree of 11 Cucurbitaceae species. Branch color of phylogenetic tree: blue, red, green and yellow represent R1-MYB, R2R3-MYB, R3-MYB and R4-MYB, respectively. Motif distribution of MYB genes in 11 accessions of Cucurbitaceae. Motif 1-6 are represented by boxes of different colors.
