# Supplementary material for: Large-scale analysis of MYB genes in Cucurbitaceae identifies a novel gene regulating plant height
Source: Hortic Res. 2025 Aug 15;12(11):uhaf210. doi: 10.1093/hr/uhaf210 (PMC12578468; doi:10.1093/hr/uhaf210)
Supplement: Web_Material_uhaf210 [file web_material_uhaf210.zip › Figure S4.pdf]

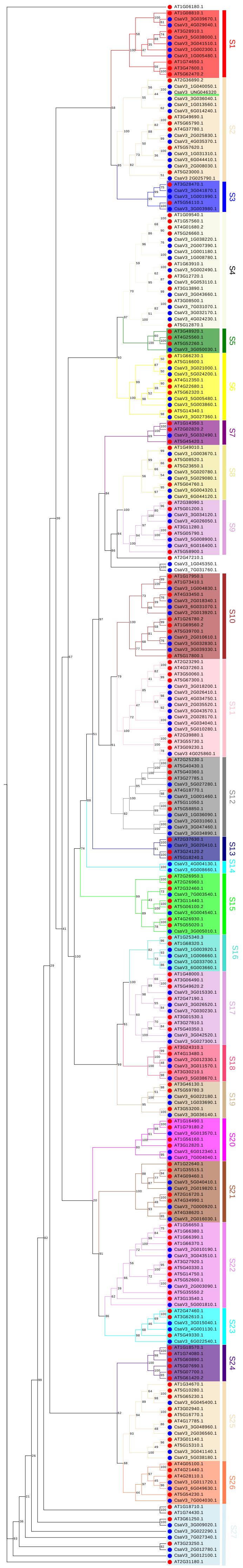

Supplementary Figure 4. The phylogenetic tree of cucumber (Chinese Long 9930) and *Arabidopsis* MYB genes, which was divided into 27 subgroups, represented by S1-S27.
